# Fabrication of multifunctional TANI/Cu_2_O/Ag nanocomposite for environmental abatement

**DOI:** 10.1038/s41598-020-70194-9

**Published:** 2020-08-21

**Authors:** Sathish Mohan Botsa, Keloth Basavaiah

**Affiliations:** 1 Department of Inorganic and Analytical Chemistry, Andhra University, Visakhapatnam, 530003 India; 2 National Centre for Polar and Ocean Research, Ministry of Earth Sciences, Goa, 403804 India

**Keywords:** Pollution remediation, Heterogeneous catalysis, Pollution remediation, Photocatalysis, Composites

## Abstract

During past decade, the ternary nanocomposite is ubiquitous in nanotechnology. Herein, a simple fabrication of cuprous oxide (Cu_2_O) and silver (Ag) nanoparticles into Tetraaniline (TANI) matrix by in situ-polymerization approach to attain Tetramer-Metal Oxide-Metal (TANI/Cu_2_O/Ag, shortly TCA) ternary composite was reported firstly. The synthesized materials were further characterized by a series of instrumental techniques to understand its structure, morphology and thermal properties. This nanocomposite showed promising applications in wastewater treatment by the testing of photocatalytic activity over the pararosaniline hydrochloride (PRA) dye degradation under visible light radiations, removal of Cadmium ion (Cd^2+^) by adsorption, corrosion resistance and antibacterial activity against both gram positive and gram negative bacterial strains. The obtained results of TCA compared with the pure TANI and binary nanocomposite (TANI/Cu_2_O) declared that the TCA composite is excellent material to solve the environmental issues due to lesser bandgap energy, visible light respond, high absorptivity, and long-life excitation.

## Introduction

Water has exploit asset on the earth, as concern for human beings, the water pollution is currently great issue throughout world and cause copious diseases almost leads to death^1,2^. In developing countries, the drinking water contamination by the second largest source mainly pesticides, chemical fertilizer residues and dyes are said to be concern agents^3^. The nanomaterials have enormous potential to deletion of heavy metals, organic pollutants, inorganic anions and bacteria have been reported^4–7^. Based on copious studies, the nanomaterials illustrate great importance for applications in purify the contaminated water.

As a result of the small size, large surface area, especially high mobility in solution, the nanomaterials ensure strong adsorption capacities and reactivity^8^. The nanomaterials exclusively metal oxide semiconductors have attracted by the scientists in evolving the pollute water treatment methods. Nanocatalysts (second class of nanomaterials) such as electrocatalysts, fenton based catalysts (photocatalysts) to developing for organic pollutants degradation and antimicrobial properties^9–11^. The number of processes has been employed yet but they are ineffective and moreover time consuming. For example, adsorption process converts the dyes from one form to another. Later the Advanced Oxidation Processes (AOP) embraces Fenton, photo Fenton, ozonisation, semiconductor based photocatalysis, photolysis have been developed for the effective decolouration of dye pollutants and destroy the microorganisms. In photocatalysis, the water and oxygen undergo redox reactions which produce extremely Reactive Oxygen Species (*OH, *O_2_ −) which are most accountable for the dyes degradation under light illumination^12^. Especially the semiconductor heterogeneous photocatalysis seems as better destructive technology to the total decolouration of dyes and destroy the microorganisms. Cuprous oxide (Cu_2_O), a p-type semiconductor and has great attention in potential applications such as solar energy conversion, photocatalysis, sensors, CO oxidation and antibacterial agents due to cost-effective, splendid physicochemical properties, high absorption activity with narrow bandgap of 2.17 eV^13,14^. But the recombination rate of electron–hole pair is very fast due to its bandgap resulting in less photocatalytic degradation efficiency.

Recently, the scientific reports have been stated that the combination of conducting polymers with metal oxide serves as alternate materials for optoelectronic applications. Among the conducting polymers, Polyaniline (PANI) has gained a lot of attention past 20 years owing to its facile synthesis^4^, chemical and environmental stability^5^ and excellent electronic properties. Therefore, PANI was used widely as conducting polymer as easy and cost-effective synthesis, fast switching redox states, metallic behaviour, and versatile switching to its conductive state through redox and protonic acid doping (with organic and inorganic acids)^15–17^. PANI has the vast applications in optical^18, 19^ electronic^20, 21^, biological fields^22,23^ and as metal anti-corrosion^24^ due to its superior conductivity and reversible re-oxidation. Tetraaniline (TANI) is aniline tetramer and has almost the same electrochemical properties as PANI^25^. However, due to its stiff structure, PANI is generally insoluble in aqueous solution, which limits its practical application^26,27^. Polymer and its nanocomposites possess unique characteristics when compared to other bulk materials due to improve the structure and controls their size range^28^. Semiconducting nature of PANI used to form nanocomposites with semiconductors (metal oxide) which are facilitating optical measurements. Though, the synthesis of monodispersed metal oxide nanoparticles into the polymer matrix of its nanocomposites with adaptable sizes and endangered from photooxidation is a major challenge. In meticulous, it is thorny to produce highly increased environmental concerns to resolve in such polymer and metal oxide nanocomposites systems. However, the silver (Ag) based composites with metal oxides gives better results in the photocatalytic efficacy by develop the charge transfer between dye molecules and metal oxides^17^.

Hence, we attempted to appraise the synthesis and characterization of TANI/Cu_2_O/Ag (TCA) ternary nanocomposite by a simple, eco-friendly and cost-effective *insitu* polymerisation method for solving the water concern issues such as pararosaniline dye degradation, removal of cadmium ions, corrosion inhibition and antibactericidal assay over the gram negative and positive bacteria.

## Results and discussion

### Formation mechanism of TANI/Cu_2_O/Ag NC

**Figure 1:**
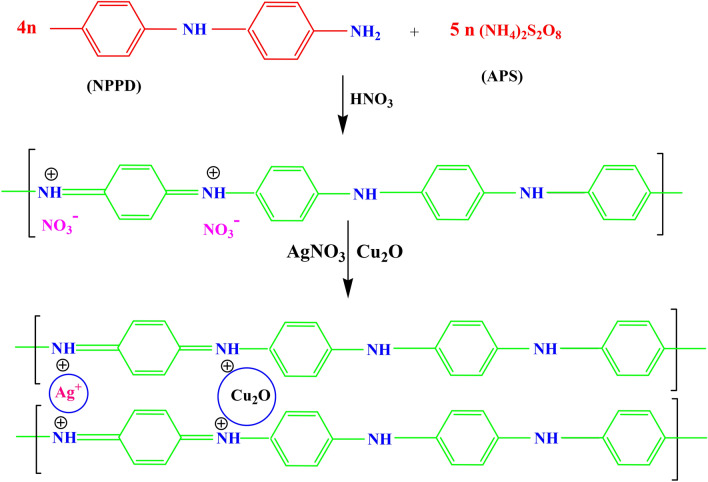
Formation mechanism of TANI/Cu_2_O/Ag Nanocomposite.

Initially, the monomer NPPD undergoes protonation by react with HNO_3_ to obtain the protonated ions in toluene medium and then the protonated ions undergo polymerization in presence of aqueous solution of APS. Here, the interjunction is formed between the toluene and aqueous media. The ions maces together and extend themselves into straight chain (1, 4 joining of phenyl groups) but the side products didn’t occur due to the fast reaction of anilinium ions. The protonation occurs on imine unit of quninonoid part of EB (TANI) and removes lone pair of electrons on nitrogen so that gets positive charge. The free NO_3_^−^ accumulates onto the EB (green colour) because of electrostatic attraction between NO_3_^−^ and TANI. The copper ions (Cu^+^) of Cu_2_O and Ag^+^ ions from AgNO_3_ which accommodate onto TANI as presented in Fig. 1.

### Characterisation study of prepared samples

#### XRD study

**Figure 2:**
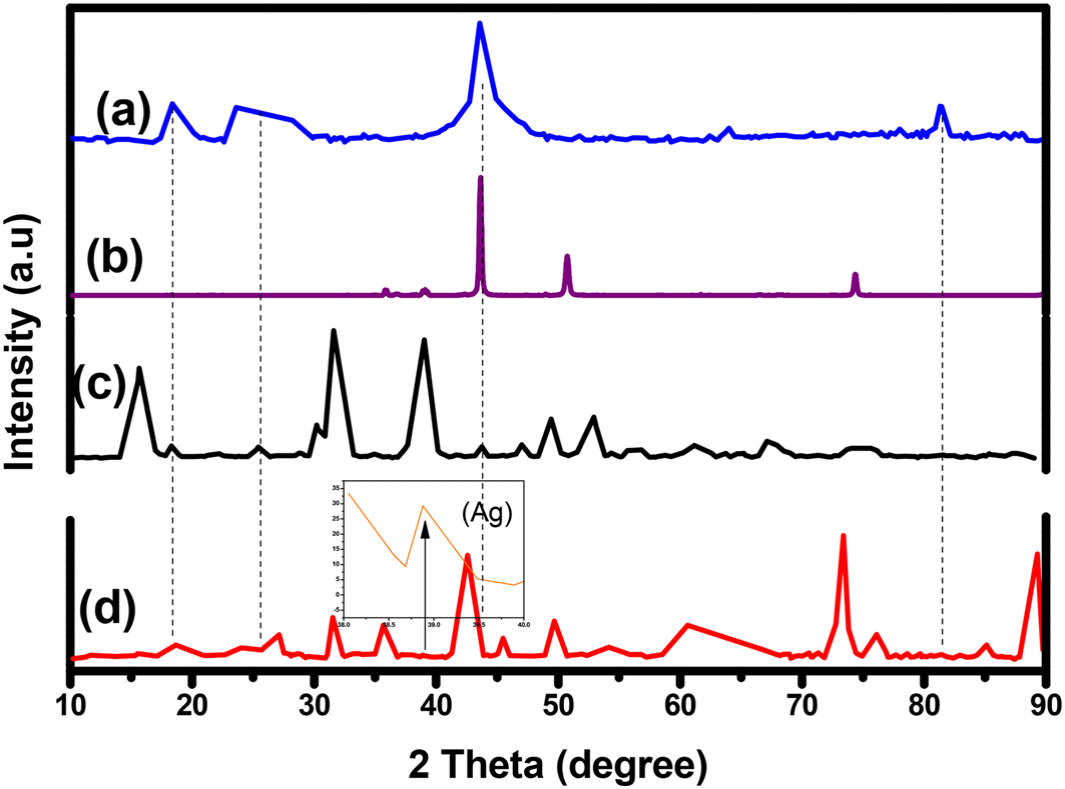
XRD patterns of (a) Cu_2_O (b) TANI (c) TANI/Cu_2_O and (d) TANI/Cu_2_O/Ag.

The pure phase formation of TANI, TANI/Cu_2_O and TCA composite was confirmed by powder XRD and the diffraction peaks of prepared materials are presented in Fig. 2. The broad peak at 2*θ* = 25° confirmed the presence of TANI^29^ along with other two major peaks at 20.6° and 27.5° due to the periodicity of parallel and perpendicular to the phenyl ring of TANI which authorizes the (110) plane and the peak was presented at 14.6° declared that formed TANI is amorphous in nature, thus XRD peaks are not sharp. Further, TANI implies four peaks have been observed in TCA NC with 2θ values of 38.4°, 44.9°, 65°and 78° which are consistent to their respective planes (111), (200), (220) and (311). The presence of sharp peak at 39°confirmed that Ag NPs are embedded in TANI/Cu_2_O/Ag matrix. The XRD pattern of Cu_2_O NPs has sharp peak at 2θ = 44° which corresponds to the (111) planes (standard JCPDS file No. 05-661) along with certain other peaks of low intensity^30^. However, these peaks show slightly shifted from their respective standard positions which may be due to the presence of Cu_2_O and Ag into TANI matrix. These results are further compiled with FTIR.

#### FTIR analysis

**Figure 3:**
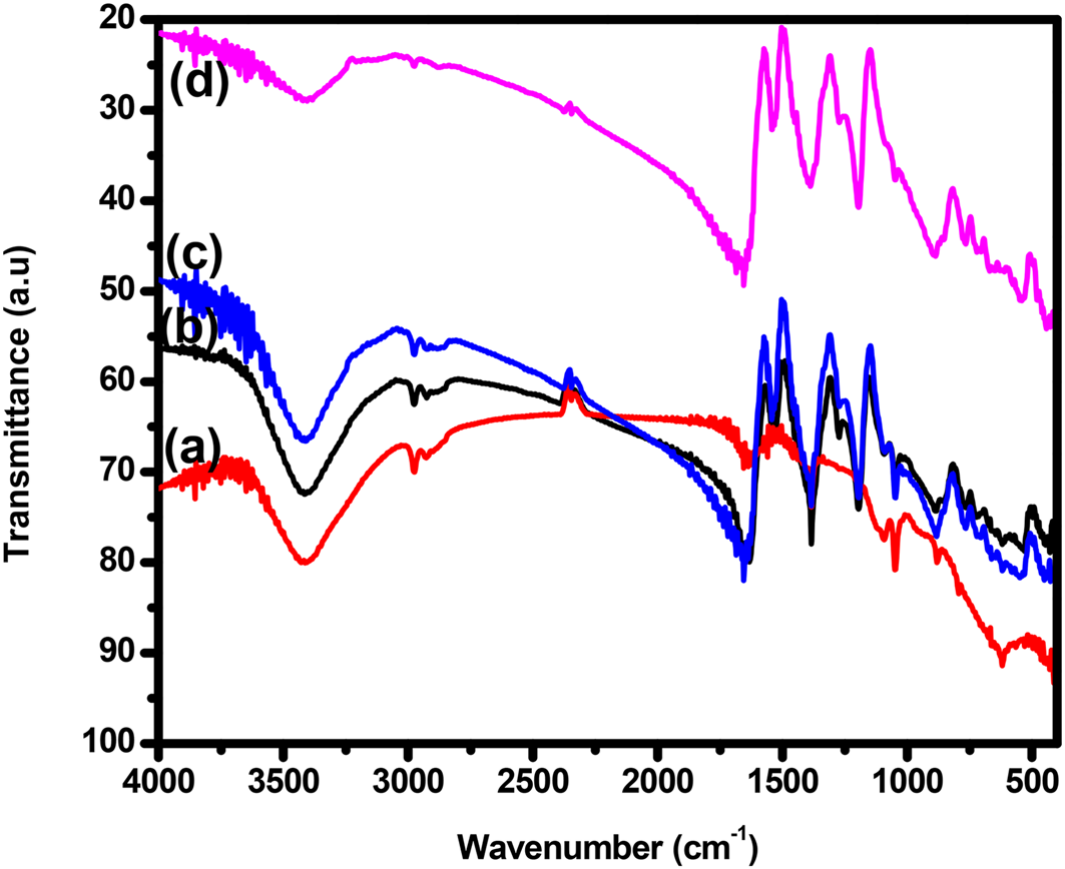
FTIR spectra of (a) Cu_2_O (b) TANI (c) TANI/Cu_2_O and (d) TANI/Cu_2_O/Ag NCs.

The FTIR spectra of synthesized Cu_2_O, TANI, TANI/Cu_2_O and TCA NCs are depicted in Fig. 3. The broad absorption peak in the range of 750–550 cm^−1^ can be attributed to C–NO_3_^−^ bonding is observed in TANI. The sharp peak appeared at 834 cm^−1^ due to N–H bending vibrations. Two peaks were observed at 1,163 cm^−1^ and 1,307 cm^−1^ could be assigned to the presence of quinonoid ring (N = Q = N) and C–N stretching mode vibrations respectively. The peaks were centred at 1581 cm^−1^ and 1,489 cm^−1^ are attributed to C–N and C–C stretching mode for quinonoid and benzenoid rings^31^. The intensities of these two peaks are almost confirms that the prepared TANI is conductive with emeraldine base (EB) form. Another peak was presented at 2,919 cm^−1^ is due to the C–H stretching vibrations whereas the peak at 3,382 cm^−1^ for the N–H stretching vibrations. These peaks were slightly shifted from their normal positions of pure TANI^32^ owing to the existence of Cu_2_O, Ag into TANI matrix.

#### FESEM–EDS analysis

**Figure 4:**
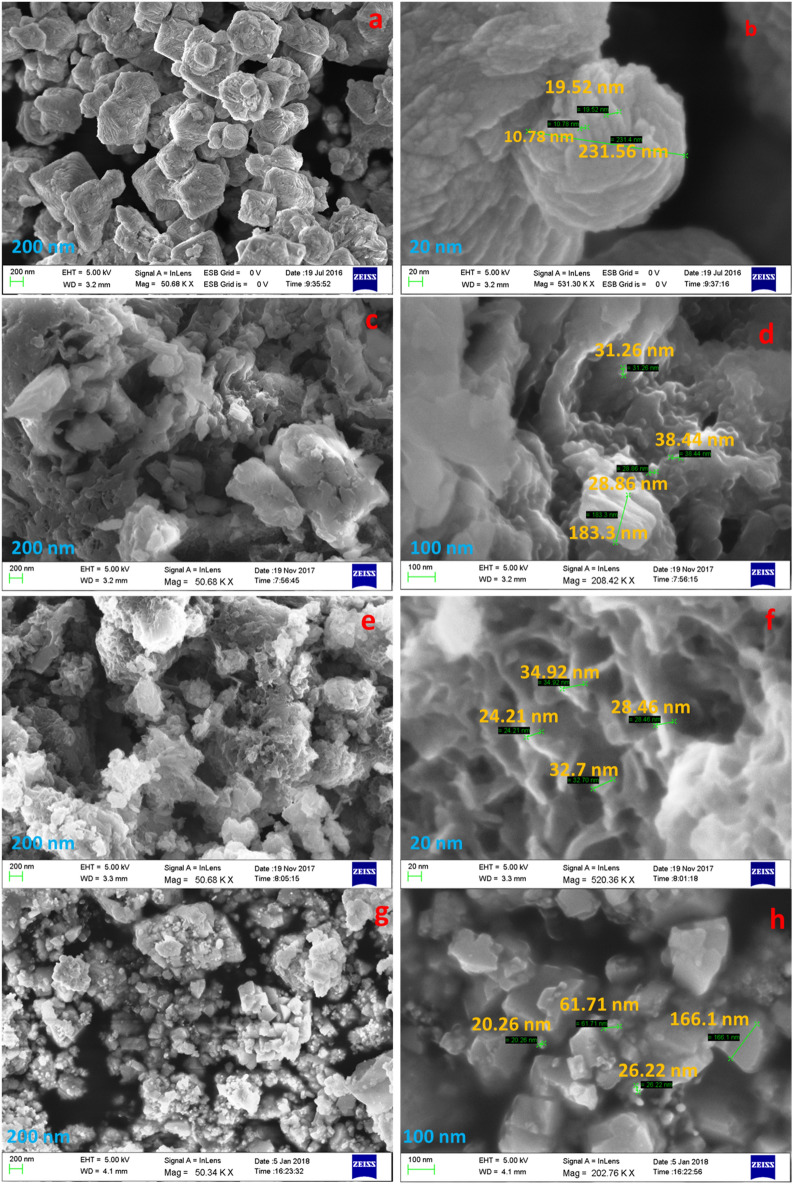
FESEM images of (**a**, **b**) Cu_2_O, (**c**, **d**) TANI, (**e**, **f**) TANI/Cu_2_O and (**g**, **h**) TANI/Cu_2_O/Ag NCs.

The morphology and structural elemental composition of prepared Cu_2_O, TANI, TANI/Cu_2_O and TCA NCs were investigated by FESEM and EDS respectively. Figure 4a,b shows the FESEM images of Cu_2_O clearly exhibited the octahedral like shape with smooth surface and an average edge length of about 478 nm. Figure 4c,d describes about the TANI, a clear and transparent sheet like structure was obtained. Figure 4e,f implies the FESEM images of TANI/Cu_2_O, in which the Cu_2_O are embedded in the matrix of TANI. It is clearly found that the surfaces of the prepared TCA NC were rough and uneven because of the presence of Ag and Cu_2_O nanoparticles are decorated on the surface of TANI as shown in Fig. 4g,h.

The presence of elements such as copper (Cu), oxygen (O), Silver (Ag), Carbon (C) and Nitrogen (N) in the EDS spectrum was confirmed the formation of pure TCA via a simple, facile *insitu* polymerization method (Fig S1).

#### Transmission emission microscopy (TEM)

**Figure 5:**
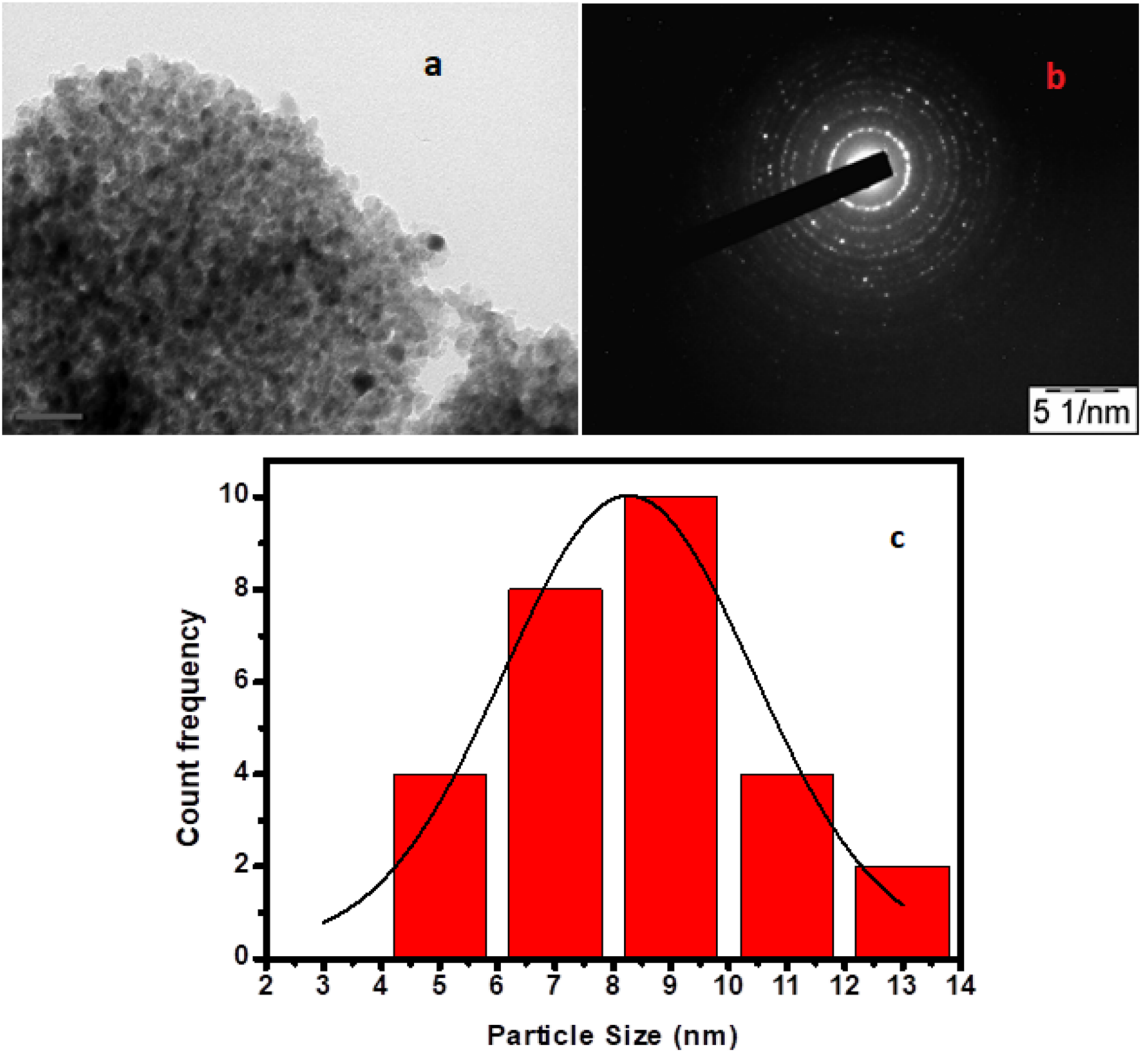
(**a**) TEM, (**b**) SAED image and (**c**) histogram of TANI/Cu_2_O/Ag composite.

Further morphology of prepared TCA NC was studied using HRTEM and Selected area electron diffraction (SAED); the images (Fig. 5a,b) indicated a poor crystalline nature of NC, supported by XRD results. It is in well agreement with the diffraction planes of TCA NC. The average particle size of TCA NC was 8.26 nm calculated from histogram (Fig. 5c) using imageJ software.

#### X-ray photoelectron spectroscopy (XPS)

**Figure 6:**
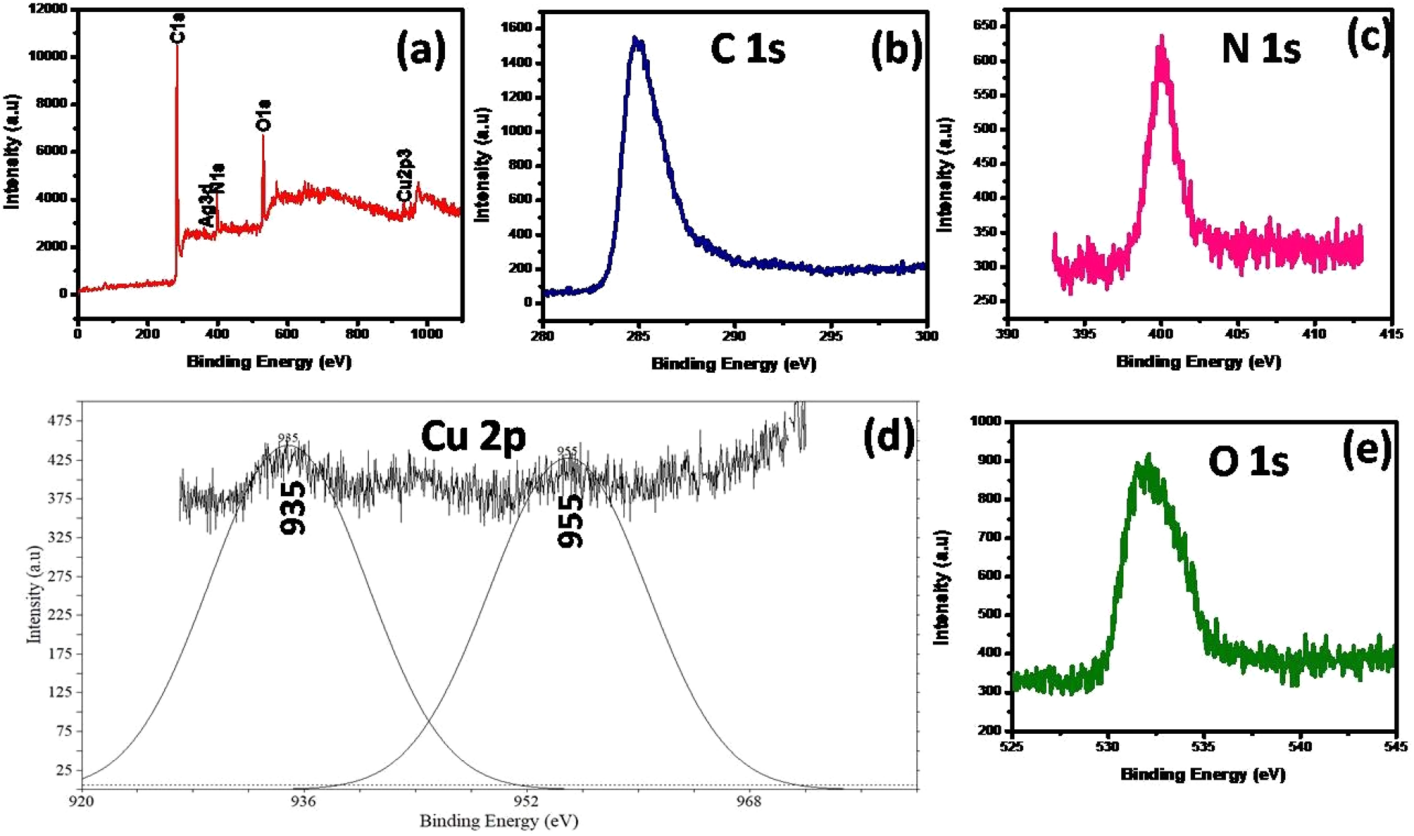
XPS analysis of (**a**) TCA NC, (**b**) C, (**c**) N, (**d**) Cu, and (**e**) O.

Figure 6 displayed the electron spectroscopy of synthesized TCA NC was examined by XPS analysis. A polymer TANI contains the carbon and nitrogen shown the respective peaks of phenyl C1s, C–C (Fig. 6b) and nitrogen containing aromatic polymers N1s (Fig. 6c) were centred at 284.8 and 399.7 eV. Besides that, the Cu and O in Cu_2_O were observed at binding energy of 934.6 eV (Fig. 6d) for Cu 2p_3/2_, 955.02 eV for Cu 2p_1/2_ and 532.2 eV (Fig. 6e) respectively^33^. When compared to Ag°, a slight peak lower shift was observed in prepared Ag which is located at 367 and 372 eV are ascribed to Ag3d_5/2_ and Ag3d_3/2_ respectively, this may be due to disturb the chemical environment around Ag NPs in synthesized composite^34^.

#### UV–visible/DRS spectroscopy

**Figure 7:**
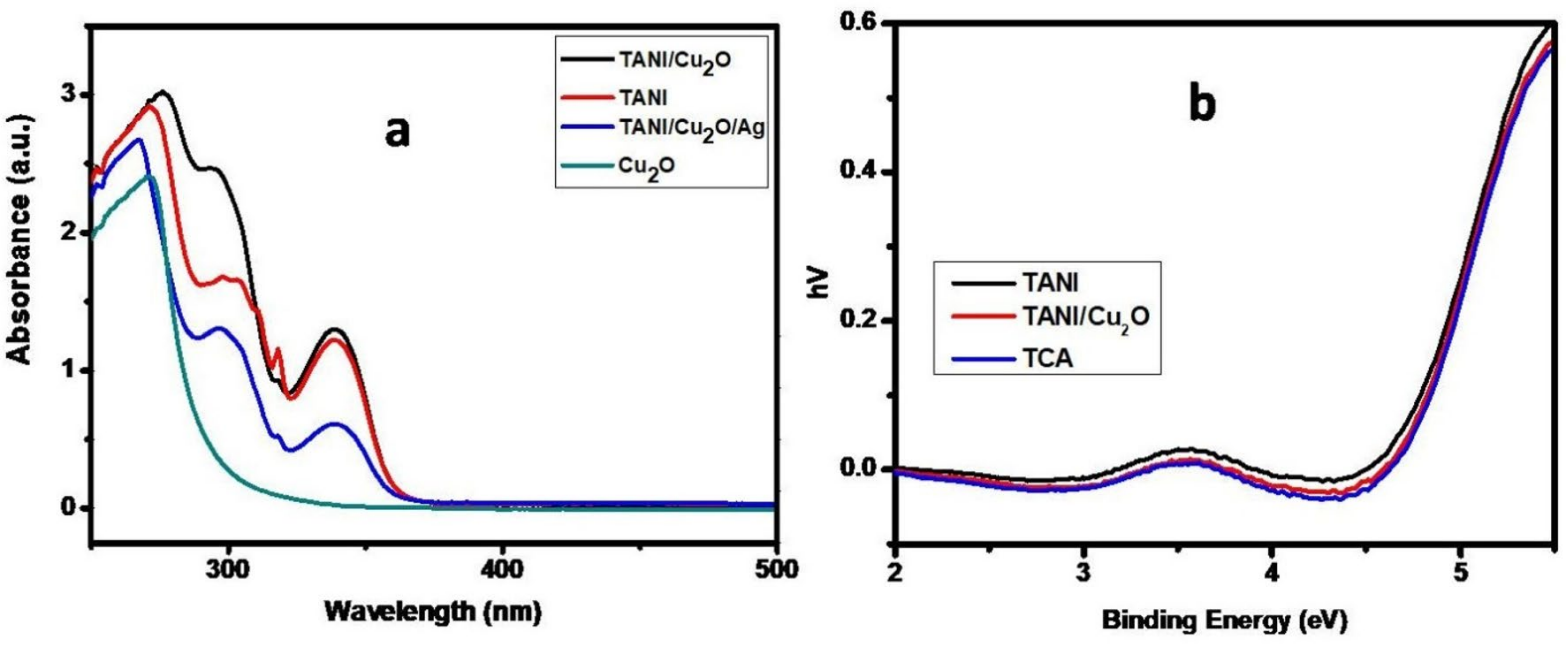
UV–Visible (**a**) absorption and (**b**) DRS spectra of prepared NCs.

The UV–Visible absorption spectra of Cu_2_O, TANI, TANI/Cu_2_O and TCA NCs are shown in Fig. 7a. The UV–Visible absorption spectra of TANI, TANI/Cu_2_O and TCA NCs show a peak at 326 nm which corresponds to π–π* transition of benzenoid ring of TANI^35^. Figure 7b (a–c) shows the optical bandgap energies of TANI, TANI/Cu_2_O and TCA NCs obtained from the Eq. (1).

1 $${\text{E}}_{{\text{g}}} = {\text{h}}\upnu = {\text{hc/}}\uplambda = {124}0{/}\uplambda$$

where ‘E_g_’ represents bandgap/forbidden energy, ‘h’ is plank’s constant, ‘ʋ’ is wave frequency, ‘c’ is the speed of light and ‘λ’ is the wavelength.

Table S1 in ESI clearly stated that the somewhat decrease in bandgap energy of TCA NC (E_g_ = 2.82 eV) from pure TANI (E_g_ = 2.93 eV) due to embedded the p-type semiconductor, Cu_2_O (E_g_ = 1.98 eV) into TANI matrix. The wider bandgap value originates from the quantum confinement effect which is caused by the nanodimensional state of materials. Lesser the bandgap materials possess excellence in catalytic activity, thus TCA NC has potent applications than other materials prepared.

#### Thermogravimetric (TG–DTA) analysis

**Figure 8:**
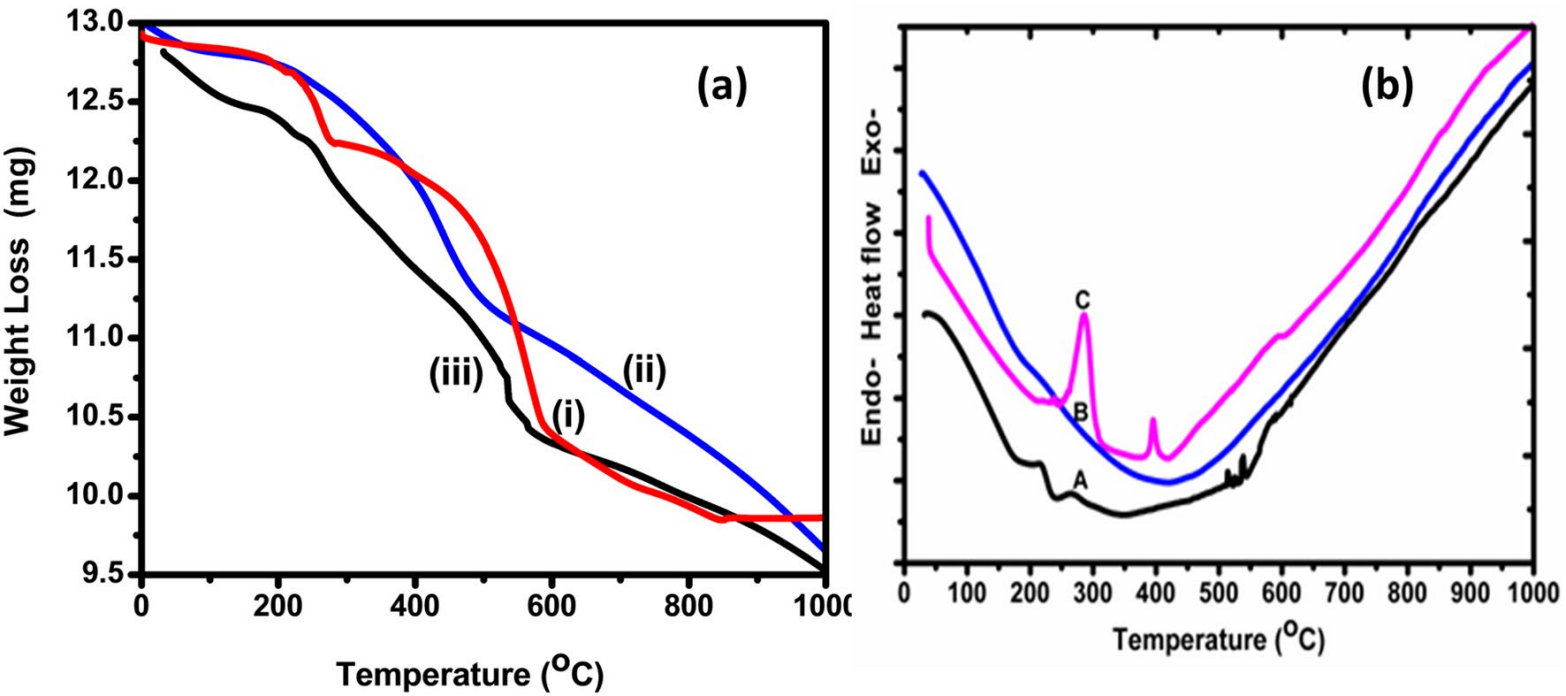
(**a**) Thermogram and (**b**) DTA curves of (**a**) pure TANI, (**b**) TANI/Cu_2_O and (**c**) TANI/Cu_2_O/Ag composites.

Thermogram of the prepared NCs in an argon atmosphere as a carrier at a flow rate of 10 °C/min with a temperature range of 30–1,000 °C is depicted in Fig. 8a. The resultant graph implies the change in mass of the sample with increase in temperature. It was observed that pure TCA NC undergo three major steps of weight loss patterns but TANI and TANI/Cu_2_O shows two major steps.

1. The first small fraction of weight loss in the temperature range from 30–270 °C is due to the loss of hydrate molecules from the surface of TANI and TCA NC held through weak ionic interactions^36^.
2. The second major step of continuous weight loss occurs due to vanish the oligomers in TANI, TANI/Cu_2_O and TCA NCs at the temperature range of 270–540°C^37^.
3. Third step is decomposition process, shows a gradual weight loss occurs in the temperature range from 600–850 °C was observed due to thermal oxidative decomposition and degradation of the polymer fraction of TCA NC^38^. From the graph, it was seen that the thermal stability of TCA NC is considerably less than that of pure TANI and TANI/Cu_2_O NC which shows high thermal stability. This may be due to decreased in chain length and the presence of bulky groups in the composites. This thermal stability was decreased in TCA NC is due to addition of Ag, may cause a decrease in the tension induced by thermal excitation in the C–C bond^39^.

Figure 8b shows the DTA curves of pure TANI, TANI/Cu_2_O and TCA NCs. This depict explored about thermal behaviour of composites from pure TANI. In Fig. 8b(C), the two exothermic peaks were observed at 280 and 420 °C, corresponding to their respective temperatures of polymer degradation and oxidation of NC. The oxidation at higher temperature means increase the content of Cu_2_O in TCA composite and is evident from the Thermogram (Fig. 8a(C)). Figure 8b(B) shows the broad endothermic peak around the temperature of 440 °C, which corresponds to the decrease in degradation temperature of TANI/Cu_2_O. Figure 8b(A) confirms the presence of exothermic peak at the temperature of 240 °C, corresponding to decomposed the TANI. These results tells about the interaction of Cu_2_O and Ag NPs with polymers surfaces lead to formation of new compound of polymers which do not decompose into simple molecules such as carbon dioxide. The decrease in the degradation temperature of polymer nanocomposite to 280 °C, as compared to 240 °C of pure TANI, indicate that the polymers in the nanocomposite is different in its chemical nature compared to that in the pure state. However, the interpretation of the result needs further investigation.

#### Photoluminescence (PL) study

**Figure 9:**
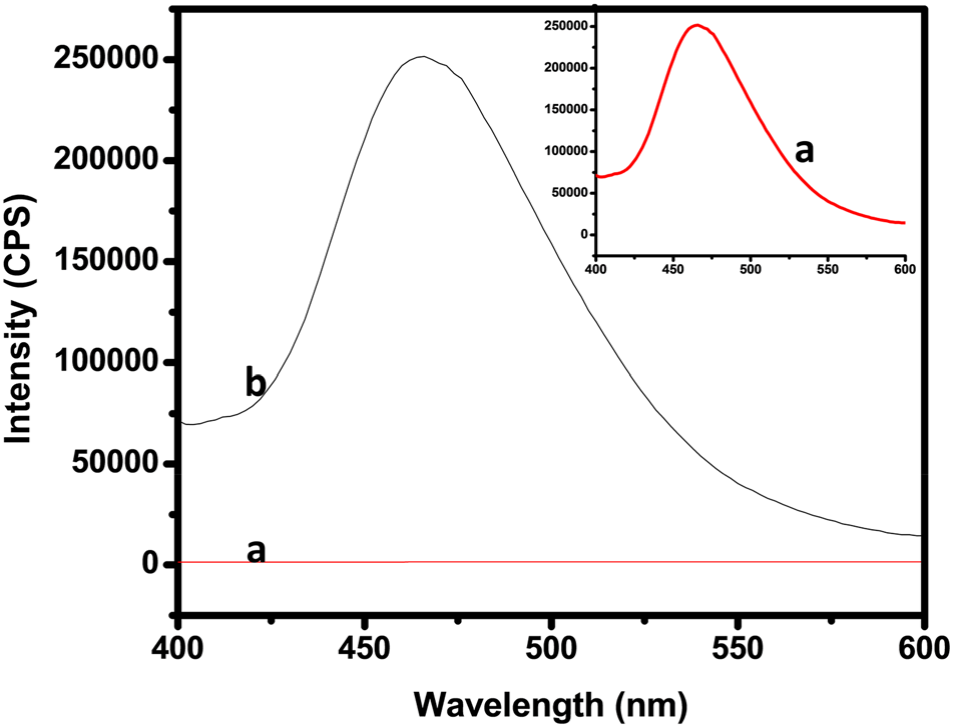
PL spectra of (**a**) TANI/Cu_2_O/Ag NC and (**b**) pure TANI.

The photoluminescence spectra used not only to characterize the defects of a semiconductor but also to study the exciton spectra in TANI and its nanocomposites. Figure 9 illustrates the emission spectra of TANI and TCA NC at the excitation wavelength of 280 nm. In order to evidence the contribution of pure TANI and TCA NC shifted to 466 nm due to transitions from the polaronic band to the -band (HOMO) structures of TANI and the emission wavelength is shifted towards the blue when compared to the bulk TANI. This emission is due to the recombination of electron and hole pairs.

### Catalytic applications of prepared nanomaterials

#### Photocatalytic activity

**Figure 10:**
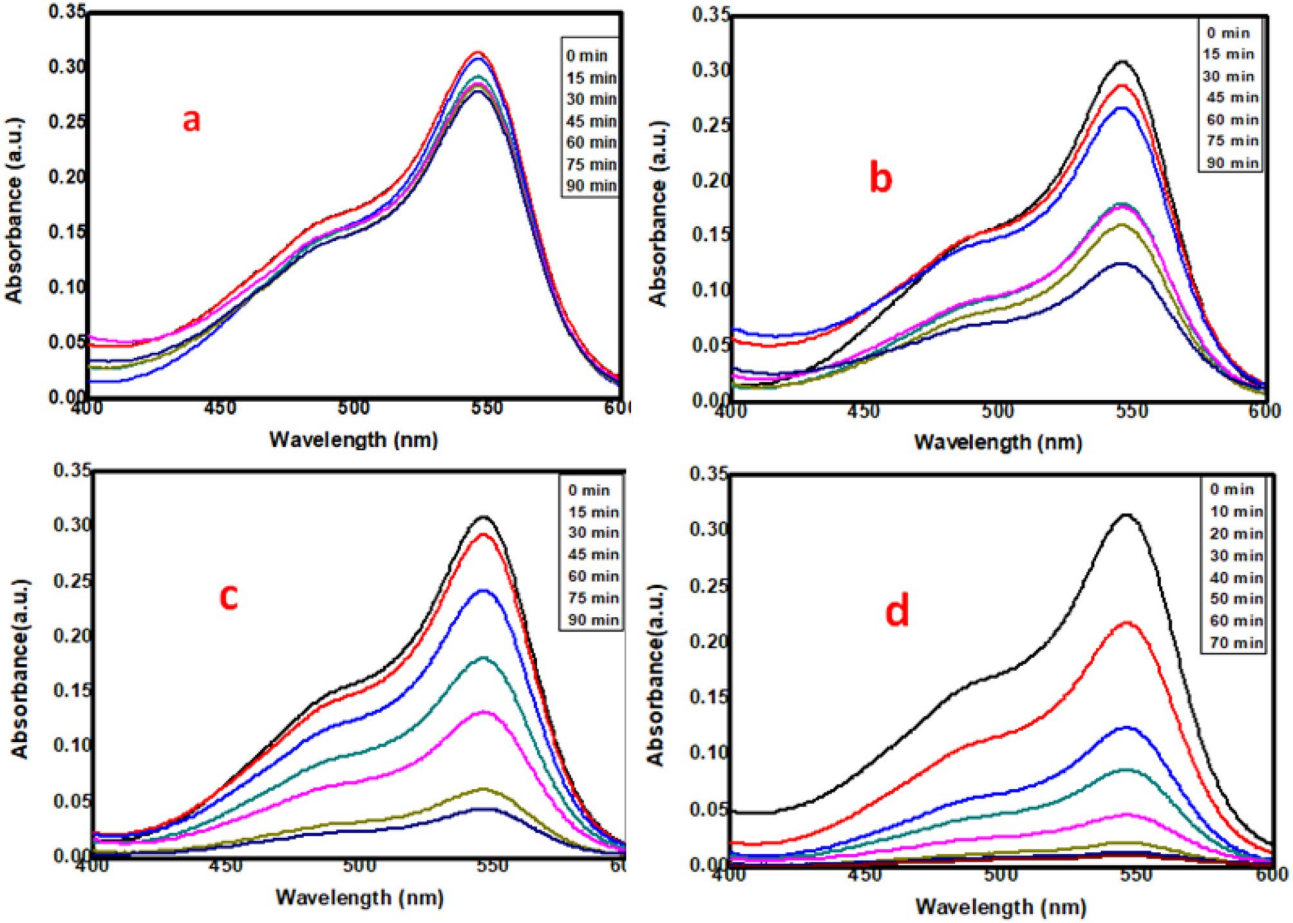
Photocatalytic activity of (**a**) TANI (**b**) Cu_2_O (**c**) TANI/Cu_2_O and (**d**) TANI/Cu_2_O/Ag.

The photocatalytic activities of TANI, Cu_2_O, TANI/Cu_2_O and TCA NCs were studied by selective organic pollutant pararosaniline (PRA) dye under visible light irradiation at normalized conditions such as dye concentration (10 mg/L), pH (11) and catalyst dose (30 mg). The characteristic absorption of PRA dye solution at the wavelength (λ = 545 nm) used to monitor the photocatalytic degradation process. Figure 10a–d shows the UV–Vis absorption spectra of PRA dye solutions by TANI, Cu_2_O, TANI/Cu_2_O and TCA composites for 90 min. These results declared that the deep green colour of the initial PRA solution gradually fades to colourless during the process of photodegradation as the exposure time is extended. Figure 10a shows the photocatalytic activity of TANI, clears that no significant photodegradation (11%) of PRA dye. Figure 10b shows the photocatalytic activity of Cu_2_O, stated that the dye was degraded partially (59%) due to faster recombination rate of electron and hole pair. Therefore, further, the binary composite TANI/ Cu_2_O was tested under same conditions and shows (Fig. 10c) the excellent photocatalytic activity (85%). So as to complete photodegradation of PRA dye, both Cu_2_O and Ag were fabricated onto TANI matrix. This ternary composite was nearly complete degraded (97%) the PRA dye (Fig. 10d) under visible light illumination in 70 min due to Ag plays an important role in photocatalysis as it entrant electrons and reduce the gap between electron and hole pair. For instance, the Ag NPs in Ag/Ag_2_O act as sink for the photogenerated electrons and contribute a great role in the phenol degradation process by preventing electron–hole recombination with holes^40^. It indicates that the ternary nanocomposite has high photostability towards the degradation of PRA under visible light illumination (Fig S2(a) in ESI). The target molecule PRA is relatively stable in aqueous solutions under visible light illumination.

In addition, the rate order kinetics of prepared composites calculated using Eq. (2), according to the Langmuir–Hinshelwood kinetics model, the function of ln(C/C_0_) versus reaction time (t) displayed a linear relationship as show in ESI (Fig S2b).

2 $$\ln {\text{C/C}}_{0} = {\text{kt}}$$

where ‘C/C_0_′ is normalized initial concentration, ‘t’ is the reaction time, and ‘k’ is the reaction rate constant (min^−1^). The corresponding pseudo first order kinetic rate constant (k) and regression coefficient (R^2^) of PRA were calculated and are listed in the Table S2 (ESI). These results confirmed that all are follows the pseudo first order rate.

### Proposed mechanism

**Figure 11:**
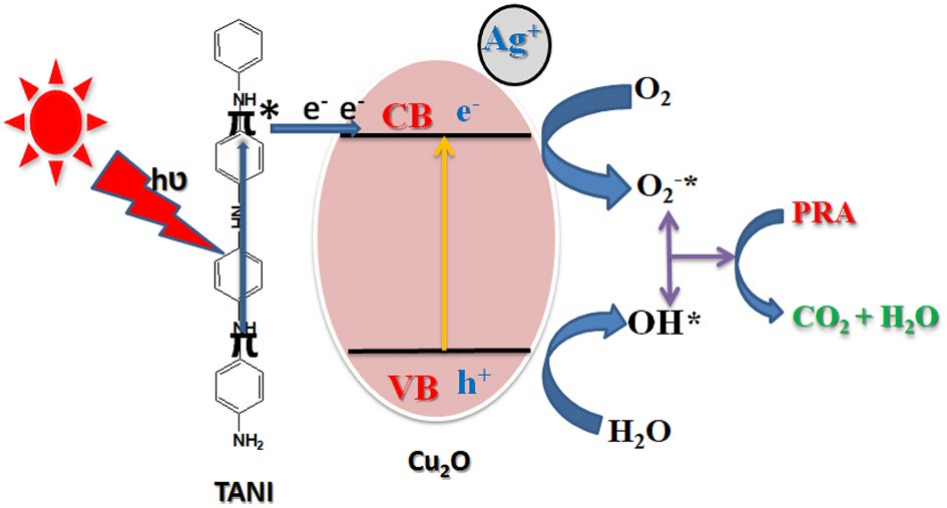
A plausible mechanism for dye degradation by TANI/Cu_2_O/Ag NC.

Proposed a plausible mechanism for improved the photocatalytic degradation efficiency of TANI/Cu_2_O/Ag ternary nanocomposite in the presence of visible light irradiation was displayed in schematic diagram Fig. 11. Initially, TANI was kept in visible light illumination, π → π * transition takes place, which release the electrons Eq. (5). In similar way, Cu_2_O undergoes charge separation process that leads to the promotion of electrons from valence band (VB) to conduction band (CB) and leaving a hole in the VB and releasing of electrons Eqs. (6, 7) due to the formation of heterojunction. Ag reduced and resulting in release of electrons Eq. (8). Finally, the photogenerated electrons are captured by the TANI through semiconductor Cu_2_O due to heterojunction which helps in enhancing the photogenerated charge separation efficiency in ternary nanocomposite. Simultaneously an equal amount of holes have been formed in semiconductor nanocomposite. These separated electrons and holes directly reacts with oxygen and water to generate highly reactive superoxide (^⋅^O_2_^−^) and hydroxyl (^⋅^OH) radicals Eq. (9). These energetic reactive radicals react with surface adsorbed PRA molecules and degraded them into CO_2_ and H_2_O Eq. (10).

Based on this mechanism, explained the photocatalytic degradation of PRA dye using TCA nanocomposite under visible light irradiation as shown by the following Eqs. (3–8).

3 $${\text{TANI}}\left( \pi \right) + {\text{h}}v \to {\text{TANI}}\left( {\pi^{*} } \right) + {\text{TANI}}\left( {{\text{e}}^{ - } } \right)$$

4 $${\text{Cu}}_{{2}} {\text{O}}\left( {{\text{h}}^{ + } } \right) + {\text{H}}_{{2}} {\text{O }} \to {\text{Cu}}_{{2}} {\text{O}} +^{ \cdot } {\text{OH}} + {\text{H}}^{ + }$$

5 $${\text{Cu}}_{{2}} {\text{O}}\left( {{\text{e}}^{ - } } \right) + {\text{O}}_{{2}} \to {\text{Cu}}_{{2}} {\text{O}} +^{ \cdot } {\text{O}}_{{2}}^{ - }$$

6 $${\text{Ag}} \to {\text{Ag}}^{ + } + {\text{e}}^{ - }$$

7 $${\text{TANI/Cu}}_{{2}} {\text{O/Ag}}\left( {{\text{e}}^{ - } } \right) + {\text{O}}_{{2}} \to {\text{TANI/Cu}}_{{2}} {\text{O/Ag}} +^{ \cdot } {\text{O}}_{{2}}^{ - }$$

8 $$^{ \cdot } {\text{OH/}}^{ \cdot } {\text{O}}_{{2}}^{ - } {\text{/HO}}_{{2}}^{ \cdot } + {\text{PRA}}\;{\text{dye}}\; \to {\text{Degradation}}\;{\text{products}}$$

Based on experimental conditions, TCA ternary nanocomposite possesses high photocatalytic activity towards the degradation of PRA dye solution under visible light irradiation than the binary composite and pure TANI.

### Radical affirmation by Scavenger test

In order to endorse the radicals scavenging, initially the 10 mg/L PRA dye solution was taken along with 30 mg TCA NC allowed to irradiation for 70 min in the presence of scavengers such as tertiary butyl alcohol (t-BuOH), Benzoquinone (BQ) for OH, O_2_^−^, and potassium iodide (KI) for hole and OH radical. Remarkably, the absence of scavenger has shown better results almost 96.3% of PRA dye suspension yet, when used scavengers declined the removal percentage like KI-40.9%, BQ-44.5%, t-BuOH-42.7% as presented in ESI (Fig S3).

### Stability and recycling of TCA composite

In favour of investigate the reusability of synthesized TCA composite, the synthesized TCA was cleaned with milli Q water followed by ethanol and centrifuged to execute the later reaction. Significantly, the photodegradation of PRA dye is declined gradually up to five successive runs as 97, 96.1, 95.2, 94 and 92.7% for the successive five cycles respectively. This may be due to the loss of TCA during washing for recyclability. Fig S4(a) in ESI stated that prepared TCA composite acted as very dynamic photocatalyst up to five successive runs, thus the TCA photocatalyst remains stable and potent because of the presence of TANI surface to Cu_2_Oand Ag in synthesized composite that leads to mobilize the photogenerated electron and holes, so enhance the photocatalytic efficacy^41^. Fig S4(b) in ESI implies the XRD patterns of TCA which confirmed that no phase change was noticed in recycled TCA composite after reusable for successive five runs. From the obtained results, we strongly declared that polymerization synthesized TCA nanocomposite can be probably used as a stable photocatalyst for the dye (PRA) degradation in large scale.

### Removal of cadmium ion (Cd^2+^) by adsorption

**Figure 12:**
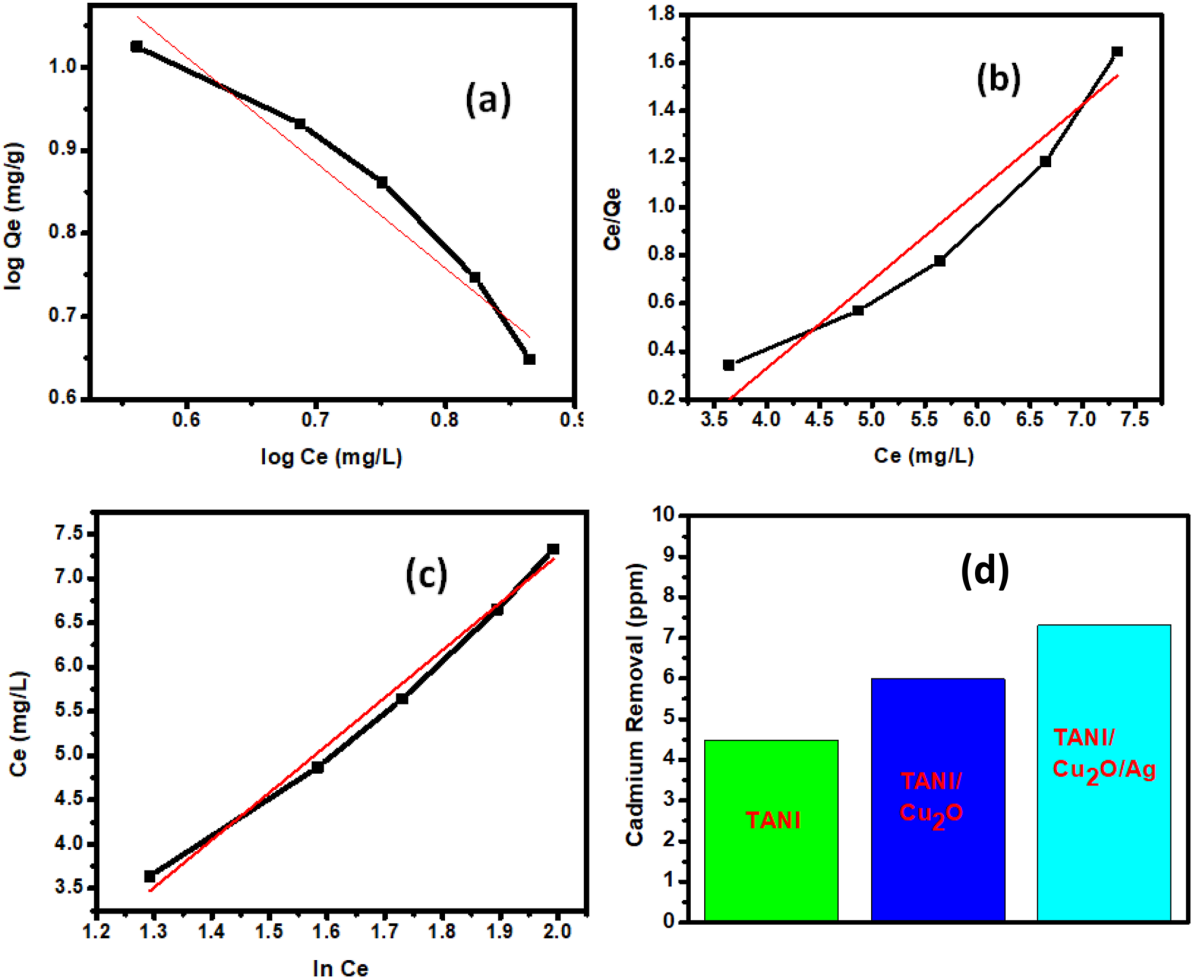
Adsorption isotherms of (**a**) Freundlinch, (**b**) Langmuir, (**c**) Temkin and (**d**) removal of Cd^2+^ (mg/L) by prepared materials.

The prepared pure TANI and their composites (TANI/Cu_2_O and TCA) were studied the adsorption efficiency by the removal of cadmium (Cd^2+^) from contaminated water under the conditions of catalyst dose 30 mg and Cd^2+^ solution 10 mg/L in alkaline medium. The obtained results declared the ternary composite shows better adsorption (7.5 mg/L) of Cd^2+^ than binary TANI/Cu_2_O (6 mg/L) and TANI (4.5 mg/L) owing to the presence of Cu_2_O and Ag NPs into TANI matrix are displayed in Fig. 12. However, the polymer based composite exhibits more adsorption capacity due to the presence of more surface groups, greater adsorption capacity, good stability and mechanic viability^42^. In this study, three most common isotherms such as Freundlich, Langmuir and Temkin models are used to interpret the experimental adsorption data. The equilibrium adsorption isotherms were determined using batch studies.

#### Freundlich model

According to Freundlich equation, the amount adsorbed increases infinitely with increasing concentration or pressure. The Freundlich Eq. (9) isotherm can be linearized into the following form:

9 $${\log}\;{\text{Q}}_{{\text{e}}} = {\log}\;{\text{k}}_{{\text{F}}} + {\text{1/n}}\;{\log}\;{\text{C}}_{{\text{e}}}$$

The regression coefficient (R^2^), slope and intercept were 0.94, 1.26 and 1.77 respectively. From these results, n and k_F_ values were calculated to be 0.78 and 58.41 respectively (Fig. 12a).

#### Langmuir model

Langmuir isotherm Eq. (10) is derived from simple mass kinetics, assuming chemisorption. This model was depicted in Fig. 12b. From this graph, calculated the regression coefficient (R^2^), slope and intercept were 0.93, 0.36 and − 1.13 respectively. From these results, Q_max_ and k_L_ are 2.73 and 0.32 respectively, desorption of the Cd^2+^ is very low.

10 $${\text{C}}_{{\text{e}}} {\text{/Q}}_{{\text{e}}} = {\text{1/k}}_{{\text{L}}} {\text{Q}}_{{\max}} + {\text{C}}_{{\text{e}}} {\text{/Q}}_{{\max}}$$

#### Temkin model

The linear relationship can be calculated from Eq. (11) and the result was depicted in Fig. 12c. The regression coefficient (R^2^), slope and intercept were calculated to be 0.98, 5.35 and − 3.44 respectively. The adsorption capacity and adsorption intensities, Q_e_ and ln k_T_; 5.9 and 0.63 respectively obtained.

11 $${\text{Q}}_{{\text{e}}} = {\text{B}}_{{1}} {\ln}\;{\text{k}}_{{\text{T}}} + {\text{B}}_{{1}} {\ln}\,{\text{C}}_{{\text{e}}}$$

From these results, the Freundlich model was fitted for the adsorption of Cd^2+^ onto ternary nanocomposite (Table 1).

**Table 1:** Rate order kinetics for adsorption isotherm models.

Model	R^2^	Intercept	Slope
Freudnlinch	0.94001	1.77388	1.26974
Langmuir	0.93305	1.13018	0.36536
Temkin	0.98716	3.44367	5.35335

### Corrosion inhibition

**Figure 13:**
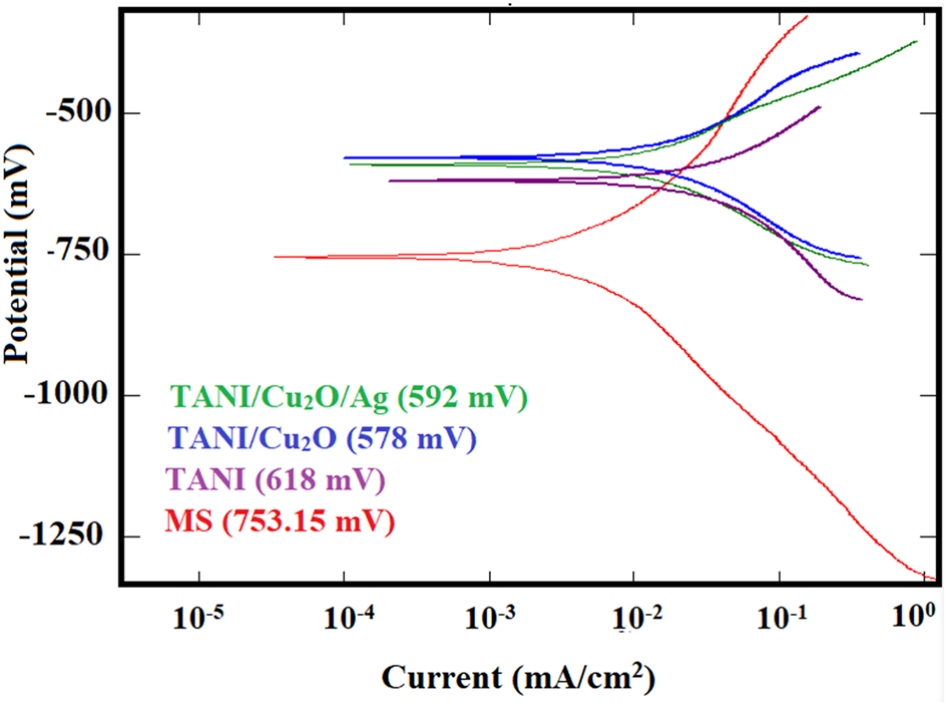
Corrosion Inhibition plot of as-prepared composites.

The corrosion resistance behaviour of synthesized pure TANI, TANI/Cu_2_O and TANI/Cu_2_O/Ag composite coatings were evaluated by sweeping the potential from equilibrium potential towards negative and positive potentials against Ag/AgCl reference electrode in H_2_SO_4_ (1 M), HCl (1 M) and NaCl (3.5%, w/w) electrolytes using Tafel polarisation and electrochemical impedance spectroscopy techniques (Fig. 13). The trends of open-circuit potential curves provided clear evidence that the incorporation of Cu_2_O into TANI nanostructures is beneficial, as it introduces potential shift towards the noble positive potential for both binary and ternary composite coatings. But the ternary composite doesn’t show the good corrosive resistance behaviour than binary composite because of low percent of Ag content in composite, which doesn’t shows the adverse effect on corrosion inhibition. Also, the corrosion resistance was enhanced with increase in the Cu_2_O content in TANI supported nanocomposite coatings. Table 2 displays the values related to the corrosion current (I_corr_), corrosion potential (E_corr_), cathodic (β_a_), anodic (β_c_) slopes and corrosion rate (CR) were calculated from tafel plots for pure TANI, TANI/Cu_2_O and TANI/Cu_2_O/Ag nanocomposites, also their polarization resistance (R_p_) was calculated from kinetic parameters obtained from Tafel plots as Stern–Geary Eq. ^43^.

12 $${\text{R}}_{{\text{p}}} = \frac{{\upbeta _{{\text{a}}} \times\upbeta _{{\text{c}}} }}{2.303} \times {\text{I}}_{{{\text{corr}}}} \left( {\upbeta _{{\text{a}}} +\upbeta _{{\text{c}}} } \right)$$

where β_a_ and β_c_ are the respective slopes of cathodic and anodic tafel plots. Fig S5 (ESI) represents the bar graph which declared that TANI/Cu_2_O binary composite has high intensity in corrosion inhibition than TANI/Cu_2_O/Ag ternary composite and pure TANI was confirmed by the values of binary composite goes towards positive of E_corr_, with the comparison of mild steel (MS) as reference. The Tafel polarization studies showed the decreased corrosion current for all the TANI/Cu_2_O composite coatings than TANI/Cu_2_O/Ag and TANI. Hence, the corrosion inhibition rate of TANI/Cu_2_O nanocomposite coatings is less than that of TANI/Cu_2_O/Ag. It is revealed that TANI/Cu_2_O nanocomposite had more corrosion resistant in 3.5% NaCl solution.

**Table 2:** Electrochemical parameters estimated from potentiodynamic polarization curves.

Specimen	E_corr_ (mA/cm^2^)	I_corr_ (mA/cm^2^)	β_a_ (mV)	β_c_ (mV)	Corrosion rate (mm/year)
TANI	− 652.46	0.07676	181.87	176.67	0.89
TANI/Cu_2_O	− 441.52	0.06121	196.16	517.53	0.71
TANI/Cu_2_O/Ag	− 616.03	0.0706	167.05	158.39	0.80

### Antibacterial assay

The antibacterial activity of as-prepared nanocomposites was tested on four different micro-organisms. For that, initially we examined with the all prepared compounds, of them, only binary and ternary nanocomposites showed better antimicrobial efficacy. Hence based on the results, further studies were on TANI/Cu_2_O and TANI/Cu_2_O/Ag NCs against the both gram negative (*Shigella flexneri*, *Salmonella* typhimurium) and gram positive (*Bacillus coagulans*, *Staphylococcus aureus*) bacterial strains at three consecutive concentrations of 40, 60 and 80 µg/mL.

#### Antimicrobial assay of TANI/Cu_2_O nanocomposite

Finally, the antimicrobial activity of TANI/Cu_2_ONC was executed against the four different pathogens including gram positive and negative bacteria. Figure S6 displays the photographic images of an inhibition zone was rendered by TANI/Cu_2_ONC against four different bacterial strains. The zone of inhibition of TANI/Cu_2_ONC for various microorganisms is shown in Table S3 (ESI). Among all the strains selected for this study, *S.aureus is* the most active strain (21 µg/mL) at the concentration of 80 µg/mL. The synthesized TANI/Cu_2_ONC is effectively inhibited the bacterial growth and gram positive (*S.aureus)* is observed to be so effective than gram negative (*S. typhimurium)* bacteria.

#### Antimicrobial activity of TANI/Cu_2_O/Ag nanocomposite

**Figure 14:**
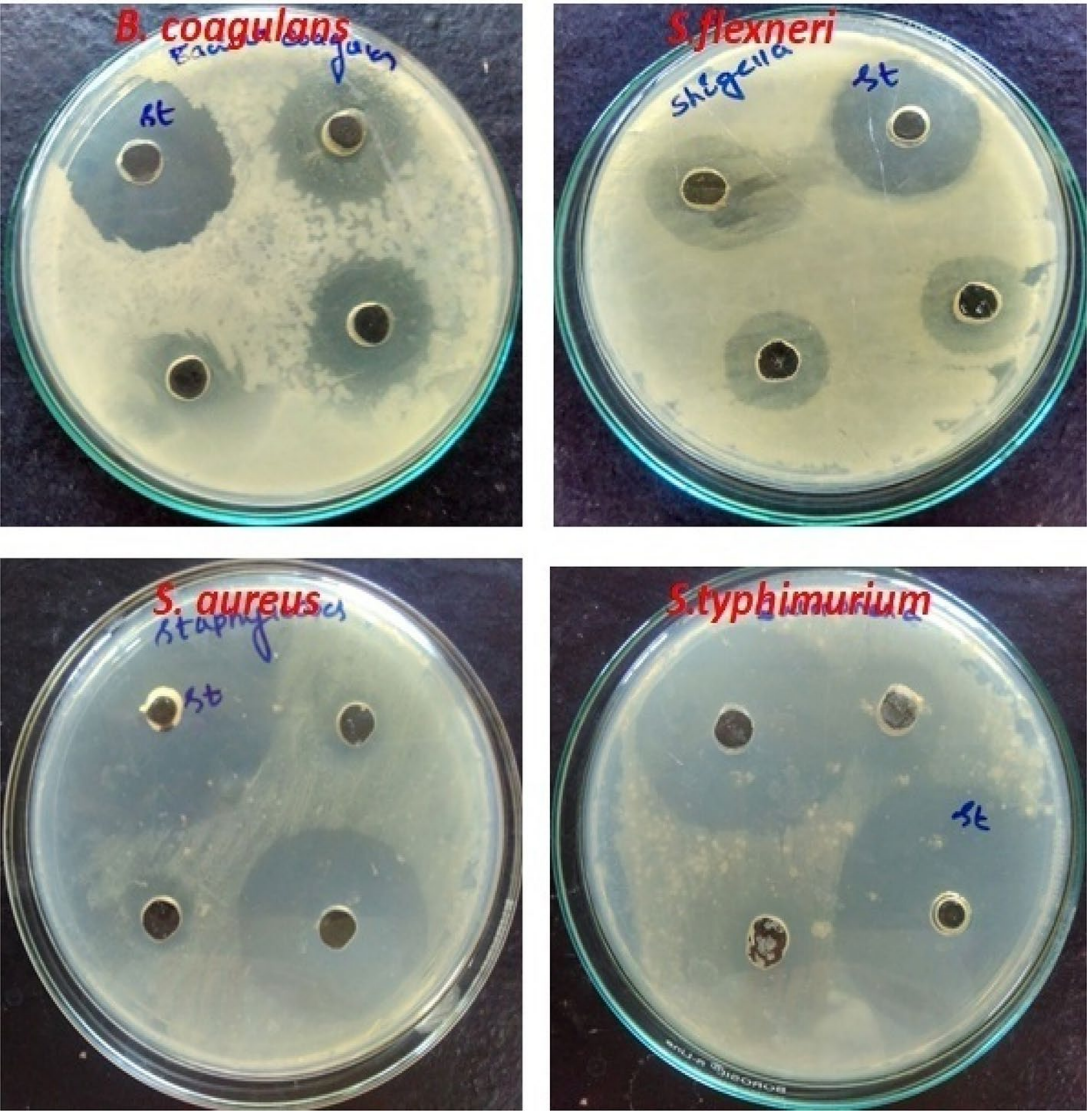
Antimicrobial activity of TANI/Cu_2_O/Ag NC.

The TANI/Cu_2_O/Ag NC was tested against the both gram positive and gram negative bacterial strains. Figure 14 shows the photographic images of an inhibition zone produced by TCA NC against four different bacterial strains. The zone of inhibition of TCA NC for various microorganisms was shown in Table 3. The influence TCA NC on *S. aureus* (25 µg/mL) and *S.typhimurium* (22 µg/mL) exhibited the best results at the examined concentration of 80 µg/mL. The prepared TCA NC effectively killing the bacterial growth and gram positive is observed to be so might effective than gram negative bacteria.

**Table 3:** The zone of inhibition of TANI/Cu_2_O/Ag NC.

S. nos.	Organism	Zone of inhibition (mm)			
		40 µg/mL	60 µg/mL	80 µg/mL	Standard (chloramphenicol) 30 µg/mL
1	*Bacillus coagulans*	16	19	20	22
2	*Shigellaflexneri*	15	16	18	20
3	*Staphylococcus aureus*	10	12	25	25
4	*Salmonella typhimurium*	12	18	22	26

The significant antimicrobial activity was not found by pure TANI and Cu_2_O. The binary and ternary composites show antimicrobial efficiency against both Gram-positive and negative bacteria. These results proposed two different mechanisms of ternary composite with target microorganism interaction, the first: adsorption of ternary composite to cell walls, membrane disruption and cell leakage which is mainly connected with high molecular weight ternary composite, the second: penetration of ternary composite into living cells leading to inhibition of various enzymes and interference with synthesis of mRNA and proteins. However, the actual mechanism of inhibition activity of TANI/Cu_2_O/Ag composite is not fully understood yet.

In present study, the antimicrobial performance confirmed that gram positive bacterial strains are more affected than gram negative (Fig S7) because Gram positive bacteria have thin peptidoglycan layer with abundant pores that permits foreign molecules to penetrate into cell and damage or malfunctioning the cell organisms. Gram positive and gram negative bacteria have differences in their membrane structure, the most distinctive of which is the thickness of the peptidoglycan layer. The high concentration of Cu^0^ affected bactericidal effect. Therefore, copper nanoparticles are chosen as a carrier in this work^44^. A very few reports on Ag ions effects on microorganisms in which Ag^+^ attracts the negatively charged cell membrane of microorganism through electrostatic interactions^45^. Hence, Ag^+^ plays a vital role in the antibacterial activity of Ag in solutions with salts, zeolites and polymers^46^. For instance, the AgNP-polymeric nanoparticles showed increased antimicrobial properties towards *E.coli* and *S.aureus*^47^. The Ag NPs induced oxidative stress on bacteria and also damaged the cell membrane, protein, and DNA^48^. However, the Ag NPs can be easily penetrate to cell wall of microorganisms and also their greater surface area enhance the antimicrobial performance by bring a huge atoms into intact with the parts of bacteria^47^. Hence, the Ag NPs were used in the study, however, they received negative charge from TANI. The biological activity of Ag and Cu nanoparticles is also dependent on their size, causing penetration and damage of Gram-positive and negative bacteria cells^49^. The mechanism of the inhibitory effects of ternary composite on microorganisms is not yet known clearly. Further investigation was required.

## Conclusion

In this work, a facile, simple and *insitu*-polymerisation approach is developed to obtain novel TANI/Cu_2_O/Ag ternary nanocomposite. On the basis of the result of UV − Vis spectra, electron density on Ag NPs results in a bathochromic shift of SPR peak of Ag NPs. Thermogram of TANI/Cu_2_O/Ag NC undergo three steps of weight loss patterns. The ternary nanocomposite was tested photocatalytic, adsorption, corrosion and antibacterial activity. It acts as benign eco-friendly material towards water treatment. It exhibits good photocatalytic activity by the complete degradation of pararosaniline (PRA) dye under visible light irradiation in 70 min. Similarly, this composite acted as good sorbent for 590 mg/g of cadmium (Cd^2+^) in 90 min. But, corrosion was inhibited more by the synthesized TANI/Cu_2_O composite than as expected TCA NC due to E_corr_ values shift towards positive and this is may be does not show any effect by Ag. TANI/Cu_2_O/Ag acts as potent and shows enhanced antimicrobial agent against both gram positive and negative bacteria. The ternary nanocomposite shows excellent catalytic properties than binary composite and pure TANI except for anti-corrosion.

## Experimental section

### Materials

Copper acetate monohydrate Cu(CH_3_COO)_2_ H_2_O, sodium hydroxide NaOH, hydrazine hydrate N_2_H_4_ H_2_O, N-phenyl-1,4-phenylediamine (NPPD), sodium chloride NaCl, hydrochloric acid HCl and silver nitrate AgNO_3_ were procured from Sigma aldrich, India. Cadmium nitrate Cd(NO_3_)_2_ and toluene were received from Merck chemicals, India. Analytical grade chemicals were utilized same as received without further purification. Throughout all synthesis process doubled distil water used.

### Synthesis of Cu_2_O Nps

For the preparation of Cu_2_O Nps^7^, initially 100 mL of 0.25 M Cu(CH_3_COO)_2_ H_2_O was ultrasonicated for 10 min, then added 100 mL of 0.1 M NaOH to the above solution. The mixture solution further ultrasonicated for 20 min, followed by adding of 1.2 mL hydrazine hydrate drop wisely and continued the ultrasonic irradiation for 30 min at ambient air. The ultrasonic irradiation was generated with a high intensity ultrasonic probe (Vibracell sonics, Ti-horn, 1.1 cm diameter, 20 kHz, 50% amplitude, 60 W/cm^2^) dipped in the reaction vessel. During the reaction, almost 50 °C temperature was recorded as not used any cooling. The reddish precipitate was formed, which is centrifuged, washed with Milli Q water and absolute ethanol in sequence, finally dried at 60 °C in hot air oven to obtain the pure Cu_2_O nanoparticles.

### Simultaneous synthesis of TANI/Cu_2_O/Ag ternary nanocomposite

In typical synthesis process, equimolar (0.01 M) of aqueous APS (adding of 10 mL 1 N HCl) and AgNO_3_ solutions were prepared with same molar of NPPD solution was prepared with toluene; as prepared 25 mL of aqueous AgNO_3_ and APS solutions were carefully added to 50 mL of NPPD solution in 250 ml beaker and then the reaction was allowed to room temperature. The aqueous phase of the solution turns into light green colour within 30 min and gradually turns into dark green colour. The beaker kept in dark room for 12 h. Then separated the aqueous phase and centrifuged. Discarded the supernatant liquid and periodical washing has been done with acetone and water. Finally the product (TANI/Cu_2_O/Ag) was dried under vacuum at room temperature. This procedure has been same for the preparation of TANI/Cu_2_O without adding of AgNO_3_ solution. Similarly, pure TANI was synthesized in the absence of both Cu_2_O and AgNO_3_ solution.

### Characterization

The diffraction peaks were recorded by powder X-Ray diffraction (XRD D8 Bruker AXS) with Cu-Kα radiation at 30.0 kV and 30.0 mA over the scan range (2θ) 10–90° at the scan rate of 2°/min. FTIR (IR Prestige21, Shimadzu Pvt Ltd, Japan) spectra of the samples in KBr pellets were obtained on a Shimadzu spectrometer in the range of 400–4,000 cm^−1^. Morphology of the prepared materials was investigated by Scanning electron microscopy (FESEM, JSM-6610LV, Jeol Asia PTE Ltd, Japan.) and the elemental analysis was done by EDS. UV–Visible absorption and diffuse reflectance spectra were recorded by UV–Visible diffuse reflectance spectrometer (Shimadzu 2600R) in the range of 200–800 nm. The PL spectra of prepared composite were taken using Flouromax-4, HORIBA, Japan to elucidate the transfer behaviour of photo-induced electrons and holes. X-ray photoelectron spectroscopy (XPS) spectra obtained from Physical electronics PHI 5,000 versaprobe III with C60 ion gun to study the electron spectroscopy of TCA composite.

### Photocatalytic studies

The photocatalytic performances of produced samples were conducted. The suspensions for the photocatalytic reaction were prepared by adding 0.03 g of various catalysts to a required concentration of dye and pollutant solutions. Before illumination, the mixture was magnetically stirred for 30 min in the dark to establish adsorption–desorption equilibrium of organic pollutants with the catalyst. A solar simulator with a metal halide lamp was used as the visible light source. The experimental solution was placed in a quartz cuvette, 100 mm away from the light source. 5 mL of the suspension was withdrawn at regular time intervals and centrifuged to remove the dispersed catalyst powder. The concentration of the clear solution was determined using UV–Visible absorption spectra. The photodegradation efficiency was determined by measuring the absorbance spectra of the dye solution before (C_0_) and after the photocatalytic reaction with variation of time (C_t_). The degradation efficiency of the photocatalytic reaction was calculated using Eq. (13)

13 $${\text{Degradation}}\;{\text{efficiency}}\;({\text{DE}}\% ) = \frac{{{\text{C}}_{0} - {\text{C}}_{{\text{t}}} }}{{{\text{C}}_{0} }} = \frac{{{\text{A}}_{0} - {\text{A}}_{{\text{t}}} }}{{{\text{A}}_{0} }} \times 100$$

where C_0_ or A_0_ are initial concentration or absorbance and C_t_ or A_t_ after the photocatalytic reaction concentration or absorbance with various time (t).

### Adsorption studies

The deletion of heavy metals like cadmium ions (Cd^2+^) via adsorption process by *insitu* polymerisation synthesized nanocomposite. During this process, 0.03 g of the sorbent was added to 10 mg/L of Cd^2+^ solution under stirring for 90 min in dark condition. Then, gathered the solution mixture at every 15 min time intervals which were further analysed by UV–Visible spectrophotometer. The amount of Cd^2+^ adsorbed onto the ternary composite can be calculated by the mass balance relationship by the following Eq. (14).

14 $${\text{Adsorption}}\,{\text{capacity}}\;\left( {\text{mg/g}} \right),\;{\text{q}}_{{\text{e}}} = ({\text{C}}_{{\text{o}}} - {\text{C}}_{{\text{e}}} ){\text{/W}} \times {\text{V}}$$

where w is the weight of the adsorbent (g), and V is the volume of the solution (L).

### Inhibition of corrosion analysis

The anticorrosion activity of as-prepared samples has been analysed for pitting corrosion and the test conducted through potentiodynamic polarization procedure using software based PAR weld electrochemical system of the GILL AC unit with the sample dimensions of 20 × 20 × 10 mm. A saturated calomel electrode and carbon electrode were used as reference and auxiliary electrodes respectively. All the experiments were piloted in aerated 3.5% NaCl solution with pH adjusted to 10 by adding potassium hydroxide. The exposure area for these experiments was 1 cm^2^ and current has been inducted into an electrolyte. The pitting corrosion resistance of the samples has been determined by obtained E_corr_ values, which is current equal to the potential at anode and cathode.

### Inhibition of bacterial growth assay

The synthesized TANI and its binary and ternary nanocomposites were tested for antibacterial activity over the bacterial strains of Salmonella typhimurium (MTCC-3231).

*Shigella flexneri* (MTCC-1457), *Bacillus coagulans* (MTCC-5856) and *Staphylococcus aureus* (MTCC-3160) grown overnight at 37 °C temperature and the medium was sterilized by autoclaving at120 °C (15 lb/in^2^). The nutrient agar medium, 20 mL with the respective bacterial strains of bacteria were transferred aseptically into each sterilized Petri plate, which were allowed to cool at room temperature for solidification. Each plate was made into 5 wells with equal distance of 6 mm sterile borer. Chloramphenicol was taken as positive control for bacterial species. The antibacterial activity of the compound was evaluated by well-established Agar well diffusion method. The four wells were made and filled with concentrations of 40, 60 and 80 µg/mL of tested samples and then plates were incubated for a period of 24 h at 37 °C in incubator, after incubation the inhibitory zone formed around the well was measured.

## Supplementary information

Supplementary Information 1.

## Data Availability

The data was generated and analyzed during the current study are available from the corresponding author on reasonable request.
