# Supplementary material for: Fabrication of multifunctional TANI/Cu2O/Ag nanocomposite for environmental abatement
Source: Sci Rep. 2020 Aug 21;10:14080. doi: 10.1038/s41598-020-70194-9 (PMC7442793; doi:10.1038/s41598-020-70194-9)
Supplement: Supplementary file 1 — Supplementary Information 1. [file 41598_2020_70194_MOESM1_ESM.pdf]

Supplementary Information

Fabrication of multifunctional TANI/Cu_2O/Ag nanocomposite for environmental applications

Sathish Mohan Botsa[a,b,*] and Keloth Basavaiah[a,]

[a]Dept. of Inorganic and Analytical Chemistry, Andhra University, Visakhapatnam-530003, India.

[b]ESSO-National Centre for Polar and Ocean Research, Goa-403804, India.

*Corresponding author: bsathish401@gmail.com

Table S1: Bandgap energy of the prepared samples

Sample	Bandgap, Eg (eV)
Cu_2O	1.99
TANI	2.93
TANI/Cu_2O	2.89
TANI/Cu_2O/Ag	2.82

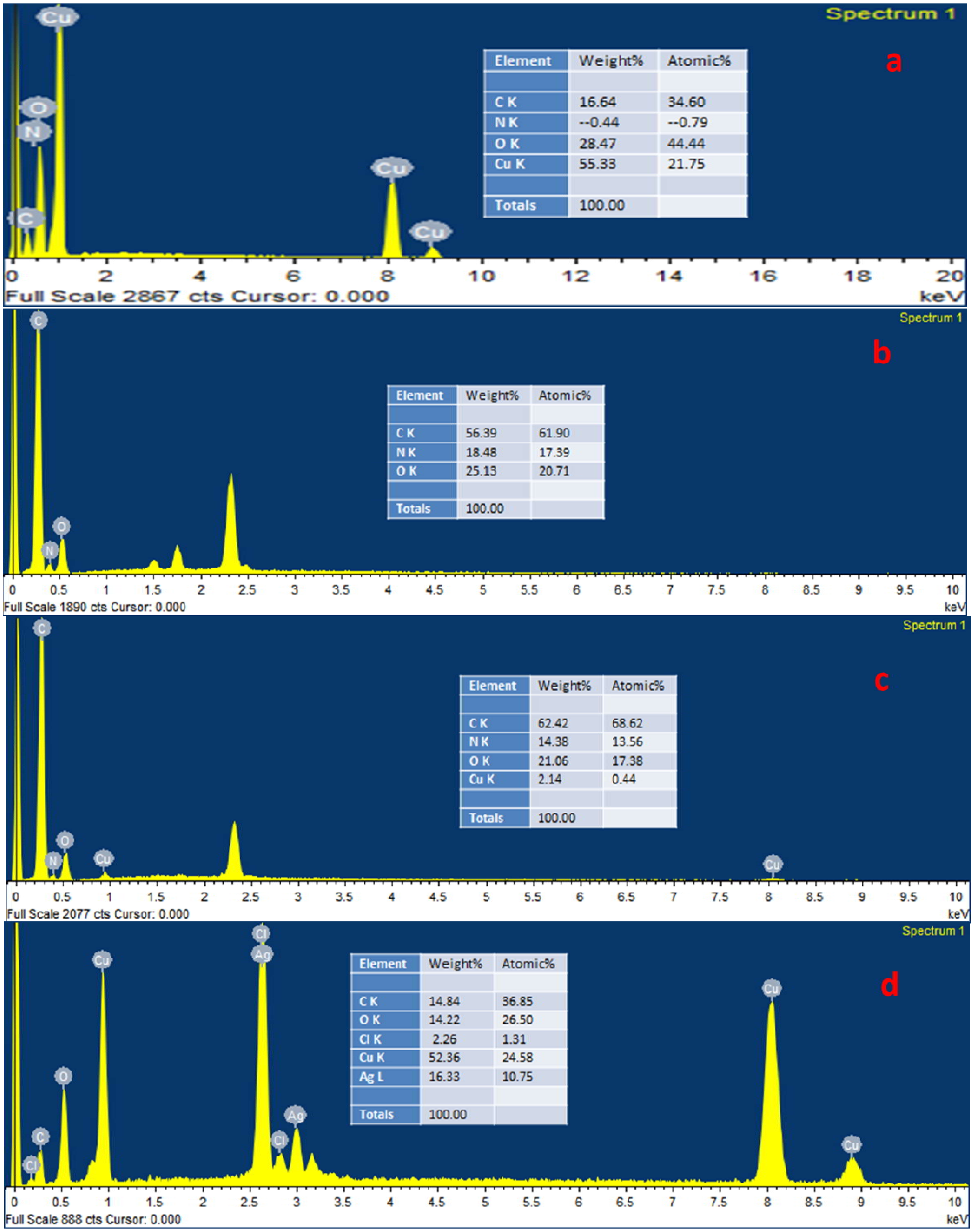

Fig S1: EDS images of (a) Cu_2O, (b) TANI, (c) TANI/Cu_2O and (d) TANI/Cu_2O/Ag NCs

Table S2: The regression coefficient (R^2) and rate order kinetics (k) of synthesised composites

**Specimen**	**R^2**	**k x 10^{-3} (min^{-1})**
TANI	0.93	1.4
Cu_2O	0.93	6.9
TANI/Cu_2O	0.98	10.6
TANI/Cu_2O/Ag	0.85	13.3

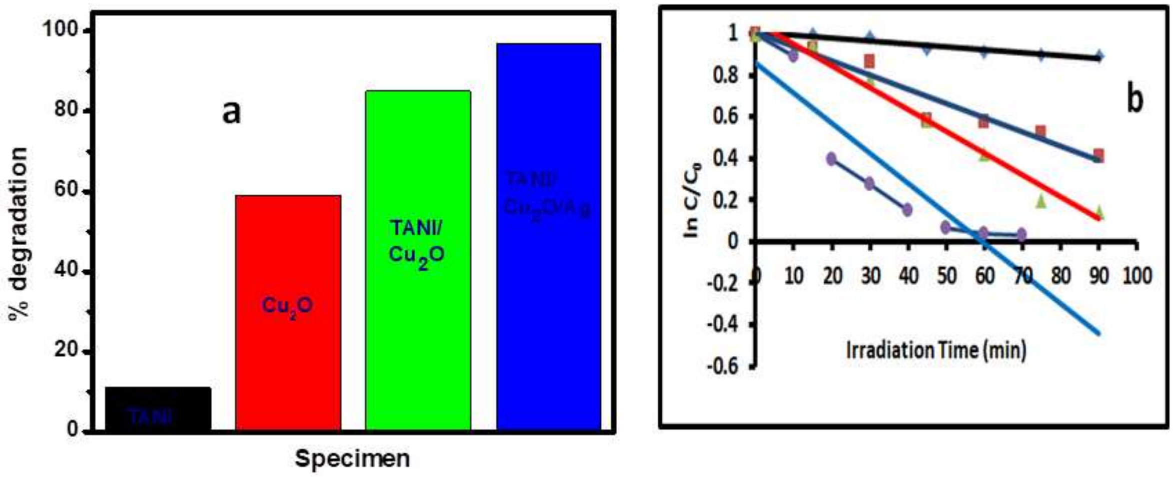

Fig S2: a) % degradation and b) kinetic rate order of prepared composites

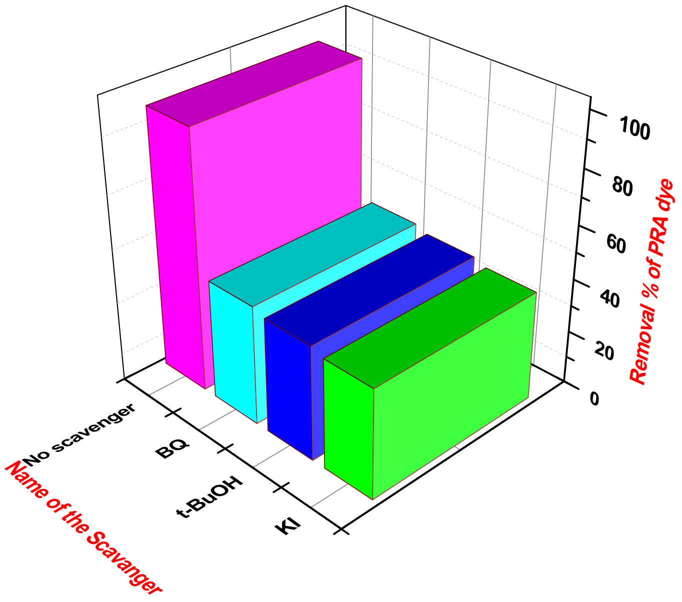

Fig S3: Scavenger test for redical confirmation

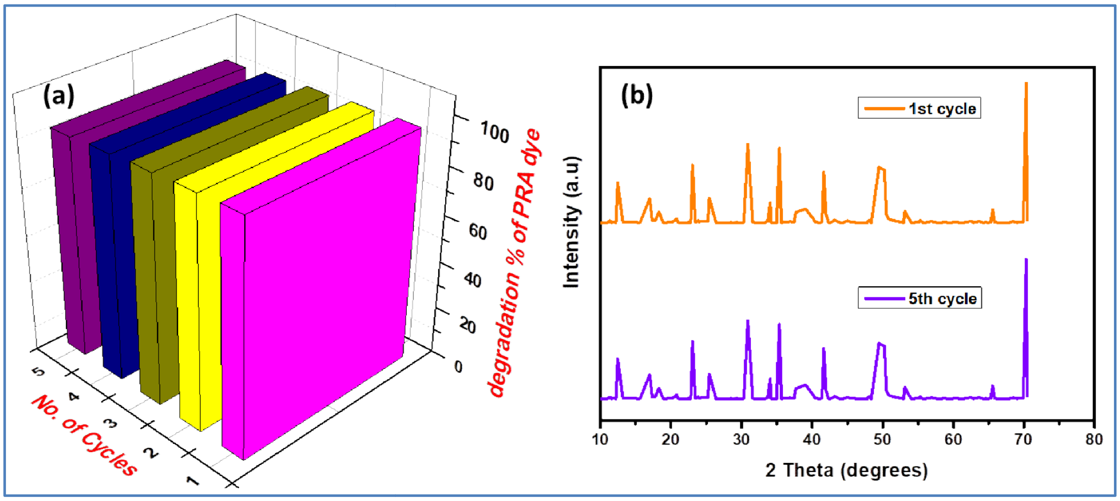

Fig S4: Recycle (a) and stability (b) of TCA NC

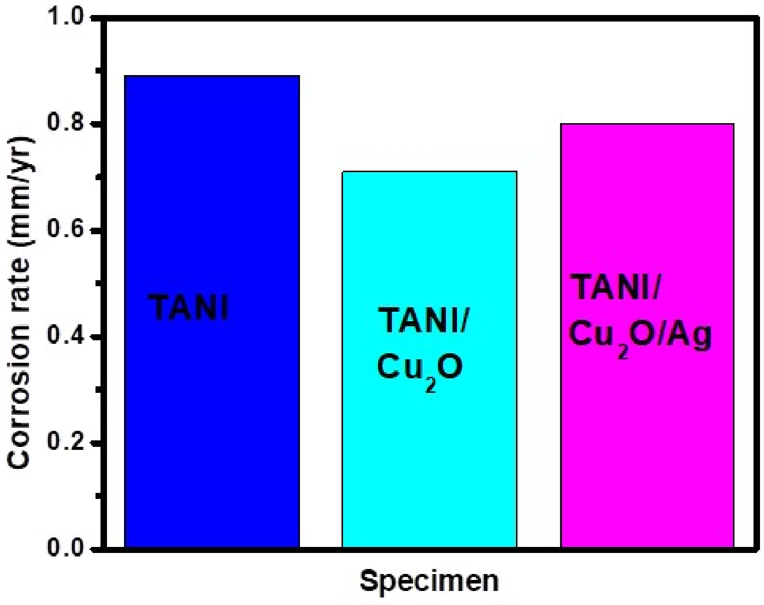

Fig S5: Corrosion rate of synthesized samples by insitu polymerization

Table S3: The zone of inhibition of TANI/Cu_2ONC

	Organism	Zone of inhibition (mm)			
		40µg/mL	60µg/mL	80µg/mL	Standard (Chloramphenicol) 30µg/ml
1	*Bacillus coagulans*	10	12	15	22
2	*Shigellaflexneri*	12	13	16	20
3	*Staphylococcus aureus*	08	10	21	25
4	*Salmonella typhimurium*	10	16	19	26

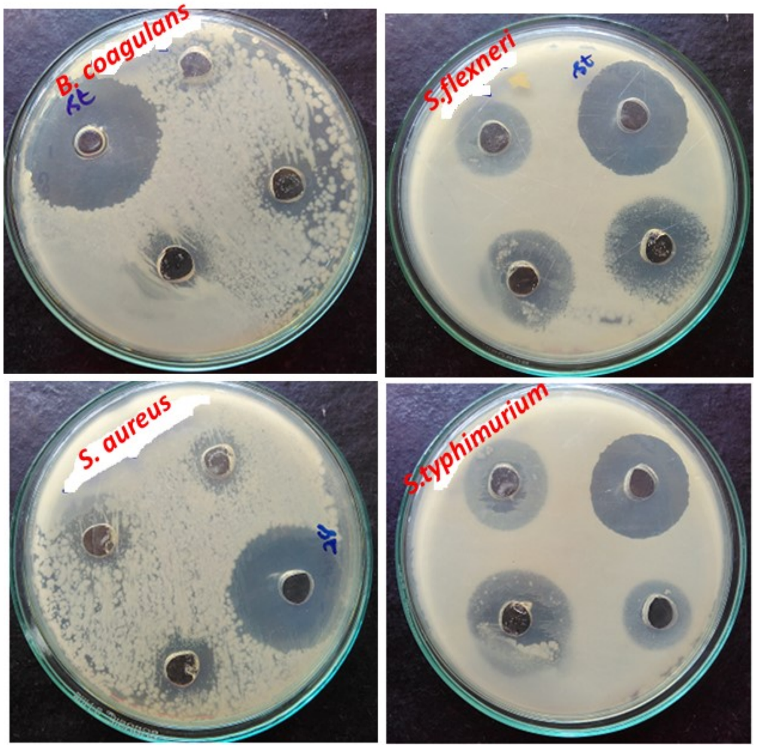

Fig S6: Antimicrobial activity of TANI/Cu_2O NC

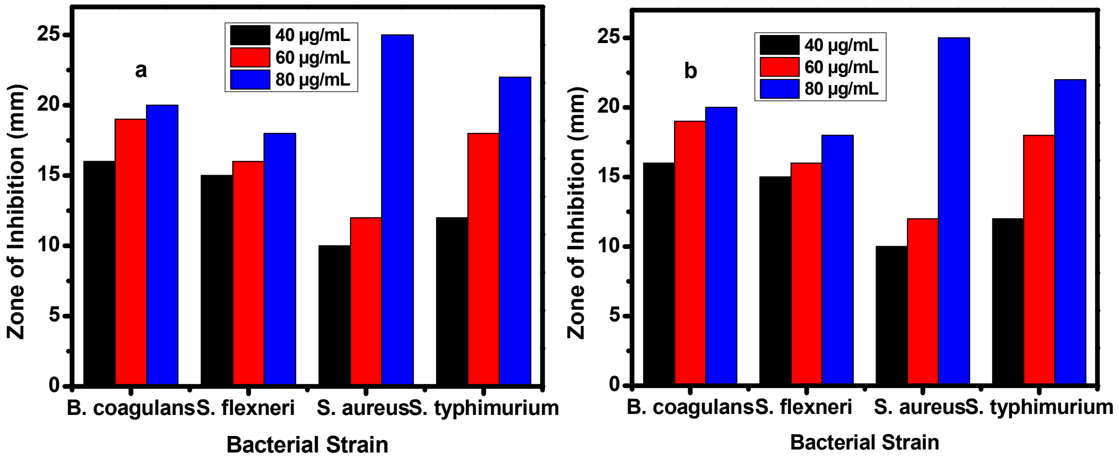

Fig S7: Zone of inhibition by a) TANI/Cu_2O and b) TANI/Cu_2O/Ag composites.
